# Noise-Driven Return Statistics: Scaling and Truncation in Stochastic Storage Processes

**DOI:** 10.1038/s41598-017-00451-x

**Published:** 2017-03-22

**Authors:** Tomás Aquino, Antoine Aubeneau, Gavan McGrath, Diogo Bolster, Suresh Rao

**Affiliations:** 1 Department of Civil & Environmental Engineering and Earth Sciences, University of Notre Dame, 46556 Indiana, USA; 2 Lyles School of Civil Engineering, Purdue University, 47907 Indiana, USA; 3 Ishka Solutions, Western Australia, Australia

## Abstract

In countless systems, subjected to variable forcing, a key question arises: how much time will a state variable spend away from a given threshold? When forcing is treated as a stochastic process, this can be addressed with first return time distributions. While many studies suggest exponential, double exponential or power laws as empirical forms, we contend that truncated power laws are natural candidates. To this end, we consider a minimal stochastic mass balance model and identify a parsimonious mechanism for the emergence of truncated power law return times. We derive boundary-independent scaling and truncation properties, which are consistent with numerical simulations, and discuss the implications and applicability of our findings.

## Introduction

Many natural and engineered systems may be conceptualized as perturbed by external noise. Examples span geology^1^, seismology^2^, forestry^3^, medicine^4^, hydrology^5^, hurricane climatology^6^, avalanche prediction^7^, ecology^8^, insurance^9^, trade^10^, and social sciences^11^, among others. In such systems under variable forcing, understanding return periods to desirable (resilience) or undesirable (risk) states is of paramount importance for design and decision making. This work addresses the following question: how much time will a state variable of interest spend away from some defined threshold, accounting for external variability in forcing?

External noise is often generated by another (complex) system. Rather than resolving and coupling both systems, it is convenient to describe this forcing as a stochastic process. In this context, First Return Times (FRTs) represent an ideal measure to answer the above question. For example, in eco-hydrology, where water-storage dynamics are controlled by stochastic hydro-climatic forcing (intermittent rainfall), FRT statistics can quantify conditions too wet or too dry and help forecast crop yields or ecosystem diversity and resilience^12^.

Theoretical predictions of mean crossing times are common^13, 14^. However, means provide limited information and ideally the complete probability density function is preferable. In many cases, distribution functions are assumed and parameterized, often employing exponential, double exponential or stretched exponential functional forms^15^. However, these distributions fail to reproduce commonly observed heavy (power-law) tails. A common and particularly simple stochastic process, Brownian motion, leads to FRTs that are heavy tailed^16, 17^, indicating that this may be the norm rather than anomalous. For heavy tailed processes, the mean, if it even exists, may not be an adequate representation of expected behaviors.

A common mechanism leading to power laws in FRT distributions is a balance leading to an equal probability of moving away from or towards the target threshold, resulting in rare but long excursions due to it being as unlikely to wander far away as it is to return. This is highlighted by the simple example of a Brownian random walker^16, 17^. Infinite power law (e.g. Lévy-stable^16, 18^), Gaussian, and exponential distributions, where (generalized) central limit theorems and Markovian properties^16, 18^ might guide theoretical approaches, often arise in theoretical studies^15^. It is well known, however, that finite-size effects (such as the presence of boundaries) can limit variability and confine the power law behavior to a range of scales. Truncated power laws (TPLs) represent a natural choice to model such systems, as they strike a balance between finite moments arising from the truncation of the process and heavy tailing characteristics. They are well founded with sound theoretical bases^18^.

Often in practice though, the precise mechanism that produces the truncation is unknown or disregarded, and the truncated distribution is proposed as an *ansatz*; this type of approach is common in Statistical Mechanics (the finite-size scaling argument^19^), and in determining first return or first passage times in bounded domains^20^. Thus, while possibly well founded, many approaches using TPLs to date are empirical in nature. Here we contend that TPLs emerge naturally in systems subjected to stochastic external forcing and therefore describe fundamental characteristics of the processes involved, rather than simply representing useful fitting distributions. We demonstrate their emergence rigorously and theoretically in a parsimonious model: a system with a fixed loss rate and stochastic input modeled as shot noise, which is an idealized representation of many of the natural and engineered systems mentioned above. From a stochastic mass balance, we formally derive exponentially truncated power law distributions for FRTs. The mechanism we identify, in contrast to boundary-induced truncation, arises from simple properties of the process itself. Our theoretical analysis is consistent with results of numerical simulations presented here.

## Conceptual Model

**Figure 1:**
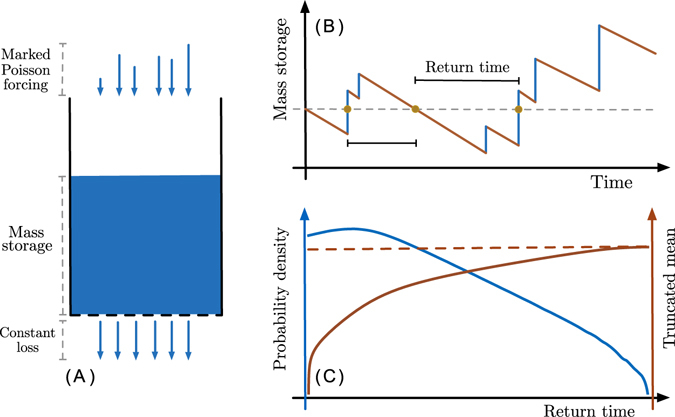
**(A)** Conceptual illustration of our mass balance model. **(B)** Illustration of the behavior of a typical trajectory for a realization of the model. Returns to a reference level (dashed horizontal line) are marked with full circles. **(C)** Typical return time probability density corresponding to a given trajectory, the mean of this density truncated to a given return time, and the full density’s mean (dashed horizontal line). The truncated mean eventually converges to the full distribution’s mean, but may differ significantly depending on the scale of interest.

In order to derive the distribution of FRTs in systems driven by external noise, we will now consider a minimal stochastic mass balance model. A schematic of the conceptual model is drawn in Fig. 1A. The model consists of a mass balance where total mass storage *M* in our system changes due to a stochastic forcing *F* and a deterministic loss *L*. Note that we use the term “mass” generically: it represents a scalar quantity stored in the system of interest. Such a model may represent, for example, a company’s supply of a particular good or the water level in a lake or wetland; similarly, forcing could represent production of the good or rainfall, while loss could describe sale of the good or leakage/evaporation. The mass balance may be written as:

1 $$\frac{{\rm{d}}M}{{\rm{d}}t}=F-L.$$

In our minimal model, we consider our stochastic forcing *F* as a Marked Poisson process, and loss *L* happens at a constant rate as long as mass *M* > 0. The Marked Poisson process represents an uncorrelated random forcing: forcing events occur at exponentially distributed intervals, and the size of each (instantaneous) event is also exponentially distributed. The choice of the exponential distribution for waiting times reflects a lack of memory: the occurrence of a forcing event has no influence on the occurrence of a future one. Correspondingly, the exponential distribution for the event size reflects lack of structure within each forcing event. As long as the typical duration of an event is short compared to inter-arrival times, the approximation of instantaneous events is reasonable. Rain for example is often modeled as a marked Poisson process^13, 21–23^. A depiction of the typical behavior of this system is illustrated in Fig. 1B. We term our model “minimal” in the sense that the forcing and loss terms are idealizations with simple properties. Thus we must highlight this simplified and idealized nature; should it be required the model must be appropriately modified for complex systems where such assumptions are unreasonable. Nonetheless this deliberately simple setup represents a limiting case of many of the more complex models used to describe the real systems mentioned in the Introduction. Considering such a minimal model enables us to pinpoint basic mechanisms in a parsimonious way, in order to aid in understanding and predicting the behavior of more complex systems.

Since the forcing *F* is stochastic, so too is *M*, and it is best described by its transient probability density *p*_*M*_, which we require to calculate FRTs. Since our system can spend finite amounts of time in a depleted state, at which there can be no loss, we explicitly account for the existence of an atom of probability *P*(*t*) at the origin and write *p*_*M*_(*m, t*) = *p*(*m, t*) + *δ*(*m*)*P*(*t*), where *δ* is the Dirac delta function^24^. With the above assumptions, taking the mean forcing event frequency to be *λ*_*t*_ and the inverse of the mean forcing event size to be *λ*_*m*_, we can write the integro-differential equation for the dynamics of the probability density (see appendix A):

2a $${\partial }_{t}p={\lambda }_{t}(E-\delta )\ast p+\alpha {\partial }_{m}p+{\lambda }_{t}EP,$$

2b $${\partial }_{t}P(t)=\alpha p\mathrm{(0},t)-{\lambda }_{t}P(t\mathrm{).}$$

The symbol * denotes convolution (in the mass variable), ∂_*x*_ denotes partial differentiantion with respect to variable *x*, *E* is the exponential density with mean ${\lambda }_{m}^{-1}$, $E(m)={\lambda }_{m}\,{\exp }^{-{\lambda }_{m}m}$, and *α* is the loss rate. This equation, often called a Master Equation, represents a probability flux balance. A generic derivation may be found in ref. 25. Specifically, early work on this model was carried out by Takács^26^, and generalizations thereof have been studied in connection with such diverse subjects as queueing theory and hydrological mass balance^13, 24, 27–32^. The full solution for *p*_*M*_(*m*, *t*) is given in appendix B. In what follows, we nondimensionalize mass variables as $m^{\prime} ={\lambda }_{m}m$ and time variables as $t^{\prime} ={\lambda }_{t}t$, dropping the primes for notational convenience. Thus, in this dimensionless framework, one unit of time corresponds to the mean time between forcing events; similarly one unit of mass corresponds to the mean forcing size. Let us also define a key critical dimensionless number $\beta ={\lambda }_{t}/(\alpha {\lambda }_{h})$, which represents the ratio between mean forcing size per unit time and mean loss per unit time.

## First return time densities

As discussed in the Introduction, truncated power law behavior has been observed in real systems^33^. When the truncation scale is long, these distributions are broad, corresponding to large variability in the return times. In such situations, the mean first return time may not be an adequate quantity for predicting a system’s behavior. Since return events longer than the observation timescale cannot be registered, studying a system at short timescales compared to its intrinsic variability results in an effective conditioning of its mean behavior to observable events. Thus, the relevant mean at a given timescale may differ significantly from the full distribution’s actual mean. This notion is illustrated schematically in Fig. 1C - note the estimated mean grows as more of the distribution is sampled. To account for this effect, we will consider full return time densities in the context of our conceptual model. Specifically, we are interested in identifying if and when power law tails are present and what determines their truncation scale. Although here we focus on first return times, our framework may also be adapted to describe first passage times where the initial and target levels differ, and to discriminate between excursions above and below the target. Our approach relies on a relationship between the (transient) probability density of eq. (2) and the first return time density *ϕ*:

3 $$\tilde{\varphi }(s,{m}_{\ast })=1-\frac{\beta }{\tilde{p}({m}_{\ast },s|{m}_{\ast })},$$

where the tilde refers to the Laplace transform with respect to time, *s* is the Laplace variable, and m_*_ > 0 is the return level of interest. A derivation of this formula, which is based on classical results for discrete random walks^34^, is provided in appendix C along with a detailed discussion.

Our starting point in identifying conditions under which power law behavior is expected is the well-known result that the first return time density for Brownian motion (a balanced random walk with no drift) decays like *t* ^−3/2^ at large times^16, 17^. This suggests looking for power law behavior in a system with equal average forcing and loss rates, i.e. no mean drift. In our conceptual model, this corresponds to *β* = 1. Indeed, for this setup we can show that return times decay like *t* ^−3/2^, irrespective of initial position. While real systems typically exhibit some degree of imbalance between forcing and loss, it is often reasonable to consider an approximate balance, which describes systems close to mean equilibrium but subject to (potentially strong) variability through fluctuations. A system that is strongly out of balance, on the other hand, is likely to be found in a completely depleted or completely saturated state, and thus be dominated by boundary effects. We will not examine this scenario in detail, but we do not expect it to lead to power law behavior, as supported by the results and discussion below.

Let us then consider a system close to mean forcing equilibrium, such that *β* = 1 + *ε*, where $|\varepsilon |\ll 1$. In this case, the return time density, at times large compared to the inverse of the average forcing frequency, behaves like:

4 $$\varphi (t,{m}_{\ast })\approx \{\begin{array}{ll}\frac{1}{2\sqrt{\pi }}{t}^{-\mathrm{3/2}}{e}^{-{\varepsilon }^{2}t\mathrm{/4}}, & |\varepsilon |{m}_{\ast }\ll 1\\  & t\gg 4{m}_{\ast }^{2}\\ \frac{1}{\sqrt{\pi }}{t}^{-\mathrm{3/2}}{e}^{-{\varepsilon }^{2}t\mathrm{/4}}\,, & |\varepsilon |{m}_{\ast }\gg 1\end{array}$$

Details on the derivation of these results can be found in appendix D. Note that when ε = 0 we recover the pure ${t}^{-\frac{3}{2}}$ power law scaling mentioned above. The critical term to our central theme, however, is the exponential ${e}^{-{\varepsilon }^{2}t\mathrm{/4}}$, which truncates the power law behavior. The characteristic truncation time is given by ${\tau }_{{\rm{trunc}}}=4{\varepsilon }^{-2}$. When *ε* = 0, *τ*_trunc_ → ∞ and no truncation occurs, but *τ*_trunc_ decreases and the truncation occurs earlier with growing *ε*. Note that a clear power law regime requires ${\tau }_{{\rm{trunc}}}\gg 1$, that is, a large number of forcing events should occur before the bias becomes relevant. Otherwise, the truncation dominates and exponential tailing ensues.

The dimensionless parameter *ε* represents the degree to which forcing and losses are out of balance. When they are perfectly in balance we expect pure power law behavior, in accordance with our results. Otherwise, one process is on average stronger than the other, resulting in an effective bias drift. For excursions in the opposite direction of the bias, the random jumps that push the state away from its target are eventually overwhelmed, resulting in the power law truncation. For times much smaller than *τ*_trunc_, the net influence of the bias is negligible and power law behavior occurs. Excursions in the direction of the bias acquire a finite probability of drifting away to infinity, or until a boundary is encountered. Thus, their contribution at late enough times is dictated by boundary effects.

Another important factor to consider is the influence of the lower boundary, as boundaries are known to induce truncation effects^17, 20^. Its influence may be felt at different timescales depending on the return level of interest. This is why we distinguish between two cases, $|\varepsilon |{m}_{\ast }\ll 1$ (return level close to the lower boundary) and $|\varepsilon |{m}_{\ast }\gg 1$ (return level far from the lower boundary). Since we do not consider a top boundary, excursions upwards of the return level still induce power law behavior, but for intermediate values of m_*_ trajectories reaching the boundary and returning may obscure this regime. It is worth pointing out that for $|\varepsilon |{m}_{\ast }\sim 1$ the far boundary approximation still holds for times much smaller than *τ*_trunc_, and the boundary effect is felt at times comparable to the truncation scale. This highlights the fact that $|\varepsilon |{m}_{\ast }$ represents a characteristic time for trajectories to reach the boundary and return to the target level.

**Figure 2:**
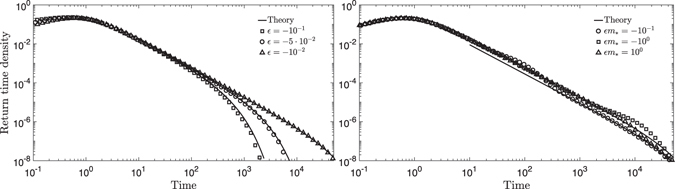
Comparison between theoretical prediction, eq. (5), and simulation results for the return time density to a given level. Simulated densities were constructed from 10^6^ subsequent return time samples binned logarithmically using 100 bins. Theoretical fits are shown starting at *t* = 10 mean forcing frequencies. **(A)** Results for different values of *ε* < 0, with high return level m_*_ so that the lower boundary effect is negligible. Results for the corresponding *ε* > 0 are similar. **(B)** Effect of the lower boundary; the two theoretical fits correspond to the cases $|\varepsilon |{m}_{\ast }\ll 1$ and $|\varepsilon |{m}_{\ast }\gg 1$.

To validate our theoretical predictions, we solved eq. (1) numerically, and sampled return times for simulations started at given levels m_*_ (the code is implemented using C++, and is available upon request). Return times were sampled by recording the time elapsed between consecutive crossings of the threshold in a single realization of the model. Because our model does not have an upper boundary, we consider excursions lasting much longer than the truncation scale as non-returning, and resume sampling for a new trajectory starting at m_*_. As explained in more detail in appendix C, we disregard (fast) crossings from above before at least one forcing event has occurred above the threshold. Comparisons are shown in Fig. 2 and the theoretical results are in very good agreement with simulations. Figure 2A shows return time densities for a reference level far from the lower boundary. This highlights the boundary-independent character of the power law scaling regime and of the truncation scale. The scaling exponent is universal for our model and analogous to well-known results for Brownian motion with no drift, and the truncation scale is proportional to the inverse of the average forcing frequency and otherwise depends only on the deviation from mean forcing equilibrium *ε*. Figure 2B illustrates the effect of the lower boundary when it is not negligible; the previous considerations remain valid, but trajectories that reach the lower boundary and return may obscure part of the power law scaling regime. Here, we do not consider an upper (saturation) boundary, but its role would be conceptually similar. If both an upper and a lower boundary are present, the width of the return time density becomes limited by the typical duration of excursions that actually reach the boundaries, which may result in an earlier boundary-induced truncation. In short, the truncation scale we identify is fully determined by the properties of forcing and loss, and it is expected to be relevant whenever boundary effects arise at longer timescales.

## Mean first return times

A typical approach to quantifying first return (or first passage) times to a given target is to describe the behavior of their mean value. This mean time can often be obtained directly, without computing the full distribution, by employing a variety of techniques available in the literature^16, 25, 34, 35^. The concept of mean first return or first passage times finds application in a variety of disciplines. Aside from the fields mentioned in the Introduction, noteworthy examples include econophysics^36^ and quantum stability^37^.

As we have argued, in the presence of broad distributions the mean alone may be of limited utility in describing the system’s behavior (recall Fig. 1C). Nevertheless, one may still obtain the mean first return time in our framework. The full expression for the Laplace transform of the first return time density, which we have obtained in closed form, contains information about all moments. Specifically, for all moment orders $n\geqslant 0$:

5 $${\mu }_{n}={\int }_{0}^{\infty }{t}^{n}\varphi (t,{m}_{\ast })\,{\rm{d}}t={(-\mathrm{1)}}^{n}{\partial }_{s}^{n}\tilde{\varphi }(s,{m}_{\ast }{)|}_{s=0},$$

where the first equality is a definition, and the second follows from the properties of Laplace transforms. As an illustration, consider the case where the threshold is close to the lower boundary, $|\varepsilon |{m}_{\ast }\ll 1$. To leading order in *ε*, one obtains for the mean and standard deviation:

6 $${\mu }_{1}={|\varepsilon |}^{-1},\sqrt{{\mu }_{2}-{\mu }_{1}^{2}}=\sqrt{2}{|\varepsilon |}^{-\mathrm{3/2}}.$$

The fact that, for small *ε*, the standard deviation is much greater than the mean may be regarded as a symptom that the mean may be insufficient as a description of the system at certain scales, since it indicates that the typical variability is much greater than the mean value.

## Discussion and Conclusions

We provide a mechanistic explanation for the emergence of truncated power law behavior in return times for systems described by a noise-driven mass balance. The specific mechanism we have identified is related to the noise itself. It exhibits a universal power law scaling exponent, and the truncation scale is determined by simple physical properties of the noise and loss processes, independent of the return level and boundary effects. While the conceptual model behind our theoretical derivation is minimalistic, we believe it highlights this generic mechanism in a clear and meaningful manner.

Our approach suggests that TPLs in FRTs should be expected when forcing and loss are close to a mean balance. If one of these is sufficiently stronger (such as for systems that are often found saturated or depleted), we expect exponential-like tailing behavior and the absence of a power law regime. It should also be noted that it is well known that boundary effects can also result in power law truncation^17, 20^. Thus, if the power law regime predicted by our modeling approach extends to scales where boundary effects (depletion and saturation) are felt, an additional truncation scale due to the boundary must be considered.

An important simplification in our model is the assumption of constant deterministic loss. While there are processes for which this assumption may be warranted, such as evapotranspiration in hydrology, this is often not the case. If at the scales of interest there is appreciable variation of the (effective) loss rate, a thorough description of the system may require a more detailed model. Although mean first passage times and steady state distributions have been reported for state-dependent loss^13, 32^, the problem is substantially more complex and we are not aware of analytical solutions for the full, transient distributions. Deterministic loss may also not be an adequate assumption for some processes, and a model accounting for stochastic effects is likely to be substantially more challenging analytically. Exploring the role of variable and stochastic loss, in particular by analyzing and comparing simulations and data for constant and variable loss, will be the subject of future work. The Marked Poisson structure assumed for the noise should likewise be regarded as a minimal assumption: it shows how unstructured noise has the capacity to generate (truncated) power law behavior. For a system where temporal or spatial forcing variability has appreciable structure at the scales of interest, additional effects may be present^17, 35, 38, 39^. A relevant case is seasonal variability, which is of particular importance in many hydrological settings^40^. We expect our results to be applicable as long as there is a separation of time scales between seasonal variability and the variability encoded by the return time distribution, but a detailed analysis of this type of scenario will be addressed in future work. Nonetheless, our results demonstrate a clear mechanism and solid foundation for TPLs as natural candidates for FRT distributions.

## Electronic supplementary material

Appendices
