# Supplementary material for: Noise-Driven Return Statistics: Scaling and Truncation in Stochastic Storage Processes
Source: Sci Rep. 2017 Mar 22;7:302. doi: 10.1038/s41598-017-00451-x (PMC5428502; doi:10.1038/s41598-017-00451-x)
Supplement: Supplementary file 1 — Appendices [file 41598_2017_451_MOESM1_ESM.pdf]

Noise-Driven Return Statistics: Scaling and Truncation in Stochastic Storage Processes

Tomás Aquino[1,*], Antoine Aubeneau[2], Gavan McGrath[3], Diogo Bolster[1], and Suresh Rao[2]

[1]Department of Civil & Environmental Engineering and Earth Sciences, University of Notre Dame, 46556 Indiana, USA
[2]Lyles School of Civil Engineering, Purdue University, 47907 Indiana, USA
[3]School of Earth and Environment, University of Western Australia, 6009 WA, Australia
[*]tdecampo@nd.edu

Appendices

Throughout the appendices, we will use the notation $\tilde{f}$ to denote the Laplace transform of f with respect to time; δ will denote the Dirac delta function; and θ will denote the Heaviside theta function. Furthermore, except for appendix A, all variables are nondimensionalized as explained in the main text.

A. Master equation for the probability density of storage

We model mass storage $\{M(t)\}_{t\geqslant 0}$ as a stochastic process assuming nonnegative real values $M(t) > 0$ for each time t. It obeys the Langevin (stochastic) equation (1). We adopt the master equation approach to describe the evolution of the probability density of $M(t)$ given the (deterministic) initial condition $M(0) = m_0$ with unit probability, $p_M(m, t|m_0) = \partial_m P\{M(t) \leqslant m\}$, where $P\{A\}$ denotes the probability of set A and is defined in the usual way [16].

The theory and method behind obtaining the master equation (2) in the main text are well established [25,27]. Here we provide a brief explanation of the meaning of each term. We account for the existence of an atom at the origin [27], $p_M(m, t|m_0) = p(m, t|m_0) + \delta(m)P(t|m_0)$. Consider first eq. (2a) for the evolution of $p(m, t|m_0)$. Essentially, the

master equation represents a probability flux balance. The term

$$\lambda_t(E * p)(m,t|m_0) = \lambda_t \int_0^m \lambda_h \exp^{-\lambda_h(m-m')} p(m',t|m_0)\,\mathrm{d}m' \tag{A.1}$$

represents increase of the probability at m due to forcing events from all possible $m' < m$. Since the forcing F is a Marked Poisson process with parameters as given in the main text, any forcing event occurs at rate λ_t; in order to obtain the rate of events from m', one must multiply by the probability density of state m', and the exponential density accounting for the probability of a jump of magnitude $m - m'$. Integrating over all possible m' results in the convolution above. The next term:

$$-\lambda_t(\delta * p)(m,t|m_0) = -\lambda_t \int_0^m \delta(m-m')p(m',t|m_0)\,\mathrm{d}m' = -\lambda_t p(m,t|m_0)\ , \tag{A.2}$$

accounts for decrease of the probability density at m due to jumps away from m, which again happen at rate λ_t. The last term:

$$\lambda_t E(m) P(t|m_0) = \lambda_t \lambda_h \exp^{-\lambda_h m} P(t|m_0) \ , \tag{A.3}$$

is similar in nature to the first, but accounts for forcing events affecting the atom at the origin.

The term $\alpha \partial_m p(m, t|m_0)$ represents deterministic loss and requires a more careful explanation. To better explain its meaning and the assumptions behind it, consider first that the loss L is a stochastic process where loss events occur at some constant rate λ_t^L, and each loss event has a magnitude $-\Delta m < 0$ with probability $g(-\Delta m)$. Then, as for the forcing F above, we have for the balance between increase and decrease of the probability density at m:

$$\lambda_t^L \int_m^\infty g(m - m') p(m', t|m_0) \, \mathrm{d}m' - \lambda_t^L p(m, t|m_0) \tag{A.4}$$

Now, deterministic loss means all events have the same size, that is $g(m - m') = \delta(m' - (m + 1/\lambda_m^L))$ for some given inverse size λ_m^L. Thus the balance above becomes:

$$\lambda_t^L[p(m+1/\lambda_m^L,t|m_0)-p(m,t|m_0)]\ . \tag{A.5}$$

If one now considers each individual event to be small, such that $\lambda_m^L \gg 1$, one may expand this result using a Taylor series to find $\alpha\partial_m p(m,t|m_0)$, where $\alpha = \lambda_t^L/\lambda_m^L$.

Consider now eq. (2b). The first term represents transfer at the loss rate α into the atom at the origin rather than to negative m. It is the correct choice to enforce conservation of the total probability for the deterministic loss described above. The second term is similar to the Dirac delta term mentioned above, and describes decrease of the atom of probability due to forcing events.

B. Solution for the probability density of storage

A method of solution for eq. (2) in the main text is provided in [29]. Its Laplace transform for the deterministic initial condition $p_M(m,0|m_0) = \delta(m-m_0)$, for some initial mass $m_0 > 0$, is given by $\tilde{p}_M(m,s|m_0) = \tilde{p}(m,s|m_0) + \delta(m)\tilde{P}(s|m_0)$, with:

$$\tilde{P}(s|m_0) = \frac{\beta}{s_m^0} e^{-s_m^0 m_0} ,$$

$$\tilde{p}(m, s|m_0) = \begin{cases} \beta^2 \frac{[s_m^0 + (s_m^0+1)s]e^{-s_m^0(m_0-m)} - [s_m^1 + (s_m^1+1)s]e^{-s_m^0 m_0 + s_m^1 m}}{s_m^0(s_m^0 - s_m^1)} , & 0 \leqslant m \leqslant m_0 \\ \beta \frac{s_m^0(s_m^1+1)e^{s_m^1(m-m_0)} - \beta[s_m^1 + (s_m^1+1)s]e^{-s_m^0 m_0 + s_m^1 m}}{s_m^0(s_m^0 - s_m^1)} , & m > m_0 \end{cases} , \tag{B.6}$$

where:

$$s_m^{0,1} = \frac{1}{2}\left[\beta(s+1) - 1 \pm \sqrt{[\beta(s+1)-1]^2 + 4\beta s}\right] , \tag{B.7}$$

where s_m^0 corresponds to the plus sign.

For comparison with [29], note that we have chosen left-continuity as opposed to right-continuity at $m = m_0$ for fixed s. The rationale behind this technical choice is discussed in detail in the next appendix section when deriving a relationship between first passage/return times and the probability density of the process.

C. First return times

We will now derive a relationship between the probability density function p of the process and the pdf ϕ of the first passage/return time. Our approach is based on classical results for discrete random walks [34], but such a relationship must be constructed with care for a continuous process, as considered here, in order to account for singular behavior. The starting point is the notion that the probability of being at $m_* > 0$ given that we have started at $m_0 > 0$ is equal to the sum of the probabilities of having returned for the first time at $t - \tau$ and then returning in a time interval $\tau > 0$, plus a (singular) initial condition contribution at $t = 0$ if $m_* = m_0$:

$$p(m_*, t|m_0) = \beta\delta(t)\delta_{m_*,m_0} + \int_0^t \phi(t-\tau, m_*|m_0)p(m_*^{\pm}, \tau|m_*)\,\mathrm{d}\tau\ , \tag{C.8}$$

where δ_{m_*,m_0} is a Kronecker delta (equal to one for $m_* = m_0$, equal to zero otherwise), and m_*^+ (limit from the right at m_*) should be used when $m_* > m_0$ and m_*^- (limit from the left at m_*) should be used when $m_* \leqslant m_0$. It should be noted that this relationship holds because the process is stationary (i.e., due to statistical stationarity of the input and output, p depends only on time intervals, and not on absolute time), and because p is

the Green's function for the process, which is described by a linear (integro-differential) equation. Since $m_*, m_0 > 0$, there is no direct dependence on the atom P, which is confined to the origin. To understand the role of the right and left limits, note that the probability of having no forcing event during time t is e^{-t}; if there is no forcing event, since both the initial condition and the loss term are taken to be deterministic, we have $p(m,t|m_0) = \delta(m - m_0 + t/\beta)$, which leads to $\tilde{p}(m_*^-, s|m_*) - \tilde{p}(m_*^+, t|m_*) = \beta$. In accordance with the previous appendix section, we take $p(m,t|m_0)$ to be left-continuous as a function of m at $m = m_0$, so that $\delta(m - m_0 + t/\beta)$ has the expected (initial condition) behavior at $t = 0$. For $m_* = m_0$, this leads to the $\beta\delta(t)$ initial condition contribution. For $m_* > m_0$ (first passage time from below), one must use $p(m_*, \tau|m_*^-) = p(m_*^+, \tau|m_*)$ in the integral contribution (initial condition just below m_*), and similarly for $m_* \leqslant m_0$ one must use $p(m_*^-, \tau|m_*) = p(m_*, \tau|m_*)$.

This singular behavior is a mathematical artifact, as the Dirac delta description is an idealization (m_0 can never be measured to infinite precision, and smooth, constant rate, deterministic loss is not a valid approximation at arbitrary scales). Nevertheless, mathematical consistency requires the regularization to ensure that our approximate description is valid even at the scales of interest.

Laplace-transforming equation (C.8) with respect to the time variable yields an ex-

plicit formula for the Laplace transform of the first passage/return time:

$$\tilde{\phi}(s, m_*|m_0) = \begin{cases} \frac{\tilde{p}(m_*,s|m_0)}{\tilde{p}(m_*^{\pm},s|m_*)} , & m_* \neq m_0 \\ \\ 1 - \frac{\beta}{\tilde{p}(m_*,s|m_*)} , & m_* = m_0 \end{cases} . \tag{C.9}$$

As above, m_*^+ and m_*^- are to be taken when $m_* > m_0$ and $m_* < m_0$, respectively. The case $m_* = m_0$ corresponds to a first return time, and in the main text and the next section we adopt the notation $\phi(t, m_*) = \phi(t, m_*|m_*)$.

We note that, since our conceptual model is mildly nonlocal in space, a jump may carry the level through a certain m_* without visiting. However, because the nonlocality is mild (the jump length probability decays exponentially), one may think of ϕ as a first crossing pdf, and assume that $p(m_*, t|m_* + \Delta h) \approx p(m_*, t|m_*)$ for probable one-jump lengths Δh. This is reasonable for time scales obeying $t \gg 1$ and $t \gg \lambda_t \Delta h/\alpha \sim \beta$, for which the noise is expected to homogenize the process over such a lengthscale. The description provided above is then valid as long as one ignores first returns from above in the absence of any forcing events, which is reasonable for $t \gg 1$. Note also that constant deterministic loss implies that a trajectory with initial condition at m_* will immediately

drop below the reference level. It is worth pointing out that real forcing events are not expected to be truly nonlocal. The conditions presented here may be seen as necessary to render the approximation of instantaneous forcing events and deterministic forcing at a constant rate reasonable and tractable with regard to the determination of first return times. Furthermore, these conditions are met in our theoretical derivations that lead to truncated power laws in the following appendix section and applied in our simulations, which are discussed in the main text.

D. Truncated power law behavior

Armed with eqs. (3) and (B.6) we can easily find an expression for the Laplace transform of the first return time density for our conceptual model:

$$\phi(t, m_*) = 1 - \frac{s_m^0(s_m^0 - s_m^1)}{\beta\{[s_m^0 + (s_m^0+1)s]e^{-s_m^0(m_0-m)} - [s_m^1 + (s_m^1+1)s]e^{-s_m^0 m_0 + s_m^1 m}\}} \quad \text{(D.10)}$$

This formula formula, however, gives us little insight. To identify late time scalings, we may use the Tauberian theorems [16] to relate large time to small s behavior. However, if a truncated power law is present, in order to identify nontrivial asymptotic behavior

a careful expansion is needed.

First note that the variation of the (ensemble) mean mass as a function of time may be obtained directly from eq. (2) by multiplying both sides by m and integrating with respect to m:

$$\alpha^{-1}\frac{\mathrm{d}}{\mathrm{d}t}\langle M(t)\rangle = (\beta - 1) + P(t)\ . \tag{D.11}$$

This shows that indeed $\beta = 1$ represents a mean balance between forcing and loss: in that case, any changes to the mean mass result from the effect of the lower boundary as encoded by the atom of probability $P(t)$. The presence of the lower boundary effectively increases the mean gains versus the mean losses, because negative mass is prevented. For the case $\beta = 1$, using eq. (3) together with eq. (B.6), we find the late-time ($t \gg 1, s \ll 1$) behavior:

$$\begin{aligned} \tilde{\phi}(s, m_*) &= 1 - \sqrt{s} + \mathcal{O}(s)\ , \\ \phi(t, m_*) &\approx \frac{t^{-3/2}}{2\sqrt{\pi}}\ . \end{aligned} \tag{D.12}$$

Following the main text, we now consider $\beta = 1 + \epsilon$, $\epsilon \ll 1$. We find:

$$s_m^{0,1} = \frac{\epsilon}{2} \pm \left(1 + \frac{3}{4}\epsilon\right) \sqrt{s + \epsilon^2/4} + \mathcal{O}(s + \epsilon^2/4) \; . \tag{D.13}$$

Substituting these expressions into eq. (D.10) we obtain:

$$\tilde{\phi}(s, m_*) \approx \begin{cases} 1 - \frac{\epsilon}{2} - \sqrt{s + \epsilon^2/4} + \mathcal{O}(s + \epsilon^2/4) \; , & |\epsilon| m_* \ll 1 \text{ and } s \ll (2m_*)^{-2} \\ 1 - 2\sqrt{s + \epsilon^2/4} + \mathcal{O}(s + \epsilon^2/4) \; , & |\epsilon| m_* \gg 1 \end{cases} \; ,$$
$$\phi(t, m_*) \approx \begin{cases} \frac{1}{2\sqrt{\pi}} t^{-3/2} e^{-\epsilon^2 t/4} \; , & |\epsilon| m_* \ll 1 \text{ and } t \gg 4m_*^2 \\ \frac{1}{\sqrt{\pi}} t^{-3/2} e^{-\epsilon^2 t/4} \; , & |\epsilon| m_* \gg 1 \end{cases} \; . \tag{D.14}$$

The case $|\epsilon| m_* \gg 1$ (return level far from the lower boundary) results from neglecting the term proportional to the exponential factor $e^{-s_m^0 m_0 + s_m^1 m}$ in eq. (B.6). Even for $|\epsilon| m_* \sim 1$, this approximation holds also for times much smaller than the truncation scale. For comparison with the $\beta = 1$ case, note that if m_* were taken to infinity in that

calculation the coefficient of $\sqrt{s}$ would be -2. However, because in that case the power law behavior for excursions above m_* is not truncated, there always exists for finite m_* a time beyond which (corresponding to a small enough s) the power law behavior arising from these excursions alone dominates.

Note that $\tilde{\phi}(0, m_*)$ corresponds to the probability of eventually returning to m_* (rather than wandering off to infinity). We have for arbitrary $\epsilon > -1$:

$$\tilde{\phi}(0, m_*) = 1 - \frac{\epsilon}{1+\epsilon}\theta(\epsilon) \ , \tag{D.15}$$

which agrees with our results to first order in ϵ. Note that in the case $\epsilon < 0$ and $|\epsilon| m_* \gg 1$ the return time probability corresponding to reaching the lower boundary and coming back is of order $|\epsilon|$ and relates to times large compared to the truncation scale, $t \gg |\epsilon|^{-2} e^{m_* |\epsilon|}$. Again, if m_* is taken to infinity, one has $\tilde{\phi}(0, m_*) = 1 - |\epsilon|$ for $\epsilon < 0$. Naturally, real systems also possess an upper boundary, so that excursions taking an infinite time to return should be interpreted as meaning the boundary is far enough away that returns happen at a different, larger time scale.
